# Supplementary material for: Surface-bound reactive oxygen species generating nanozymes for selective antibacterial action
Source: Nat Commun. 2021 Feb 2;12:745. doi: 10.1038/s41467-021-20965-3 (PMC7854635; doi:10.1038/s41467-021-20965-3)
Supplement: Supplementary file 3 — Reporting Summary [file 41467_2021_20965_MOESM3_ESM.pdf]

nature research

Corresponding author(s): Yujie Xiong, Lihua Yang

Last updated by author(s): Dec 9, 2020

Reporting Summary

Nature Research wishes to improve the reproducibility of the work that we publish. This form provides structure for consistency and transparency in reporting. For further information on Nature Research policies, see our Editorial Policies and the Editorial Policy Checklist.

Statistics

For all statistical analyses, confirm that the following items are present in the figure legend, table legend, main text, or Methods section.

n/a	Confirmed	
☐	☒	The exact sample size (n) for each experimental group/condition, given as a discrete number and unit of measurement
☐	☒	A statement on whether measurements were taken from distinct samples or whether the same sample was measured repeatedly
☐	☒	The statistical test(s) used AND whether they are one- or two-sided<br>*Only common tests should be described solely by name; describe more complex techniques in the Methods section.*
☒	☐	A description of all covariates tested
☒	☐	A description of any assumptions or corrections, such as tests of normality and adjustment for multiple comparisons
☐	☒	A full description of the statistical parameters including central tendency (e.g. means) or other basic estimates (e.g. regression coefficient) AND variation (e.g. standard deviation) or associated estimates of uncertainty (e.g. confidence intervals)
☐	☒	For null hypothesis testing, the test statistic (e.g. F, t, r) with confidence intervals, effect sizes, degrees of freedom and P value noted<br>*Give P values as exact values whenever suitable.*
☒	☐	For Bayesian analysis, information on the choice of priors and Markov chain Monte Carlo settings
☒	☐	For hierarchical and complex designs, identification of the appropriate level for tests and full reporting of outcomes
☒	☐	Estimates of effect sizes (e.g. Cohen's d, Pearson's r), indicating how they were calculated

Our web collection on statistics for biologists contains articles on many of the points above.

Software and code

Policy information about availability of computer code

Data collection	
Data analysis	Origin 8.0, Excel 2013, and Nano Measurer Software.

For manuscripts utilizing custom algorithms or software that are central to the research but not yet described in published literature, software must be made available to editors and reviewers. We strongly encourage code deposition in a community repository (e.g. GitHub). See the Nature Research guidelines for submitting code & software for further information.

Data

Policy information about availability of data

All manuscripts must include a data availability statement. This statement should provide the following information, where applicable:

- Accession codes, unique identifiers, or web links for publicly available datasets
- A list of figures that have associated raw data
- A description of any restrictions on data availability

Provide your data availability statement here.

Field-specific reporting

Life sciences study design

All studies must disclose on these points even when the disclosure is negative.

Sample size	For bacterial and cellular assays, each trial was carried out in triplicate, and the reported results are averages of two independent trials. This is a protocol commonly used in the literature (Proc. Natl. Acad. Sci. U.S.A., 2008, 105, 20595; J. Am. Chem. Soc., 2007, 129, 12141; ACS Nano, 2014, 8, 10414-10425). With each trial performed in triplicate, results from each trial are reliable to acceptable extent. As the reported results are averages of two independent trials, the reproducibility can get confirmed.<br><br>To assess the in vivo antibacterial performance and potential safety of AgPd nanocages, we constructed a skin wound-infected model with twelve ICR female mice. At 48 h after infection, the mouse models were randomly divided into 2 groups (n = 6 in each group) and then treated with AgPd0.38 or PBS, respectively. Through the observation window, we for each treatment group not only took photographs for the wound of each mouse but also collected blood samples from three mice that were randomly selected on any pre-designated specific blood collection time for monitoring the serum levels of three pro-inflammatory cytokines that are vital to inflammatory disease initiation and progression. We believe that, in this way, the results on the serum levels of these pro-inflammatory cytokines could be of acceptably good reliability and reproducibility. After the observation window was over, all mice were sacrificed, for each treatment group, wound tissues of two randomly selected mice were stained for hematoxylin and eosin (H&E) tissue staining effect analysis while those of the other four were homogenized for subsequent counting on bacterial colony forming units (CFU) per wound. As the difference in efficiency for promoting wound healing between different treatment groups had already been tracked with photographs taken for wounds through the observation window and H&E is a qualitative analysis, we used two mice randomly selected from a treatment group as representatives for that group, to ensure reproducibility. As CFU counting is a quantitative analysis and whether or not our nanocage could promote disinfection in the complex environment as in a bacterial-infected wound is unknown, we for each treatment group used the other four mice to do the CFU counting analysis, in efforts to achieve results with acceptably good reliability and reproducibility.<br><br>To assess the in vivo antibacterial performance and potential safety of Ca/PDA/AgPd, we constructed a skin wound-infected model with fifteen ICR female mice. Prior to the bacterial infection, we randomly selected five mice and collected their blood samples, to get an information on the serum levels of white blood cell and neutrophil in healthy mice. At 24 h after infection, we randomly selected five mice and collected their blood samples, to get an information on the serum levels of white blood cell and neutrophil in bacterial-infected yet untreated mice. We trust that five mice randomly selected out of 15 mice can provide reliable and representative results. At 48 h after infection, the mouse models were randomly divided into 3 groups (n = 5 in each group) and subsequently treated with PBS, Ca/PDA, or Ca/PDA/AgPd, respectively. Through the observation window, we took photographs on the wounds of all mice and, for each treatment group, collected blood samples of all mice that were still alive (it should be noted that one mouse from the PBS treatment group and one mouse from the Ca/PDA treatment group died after group allocation on day 0 but before blood sample collection on day 1), which should be reliable. At the end of the observation window, we from each treatment randomly selected two mice H&E staining analysis on wound tissues and monitored the CFU counting for wounds of the rest mice. Because the major point of this animal study is to examine whether coating a catheter with AgPd0.38 enables the otherwise inactive catheter to inhibit biofilm formation after an extended exposure (spanning 5 days) and whether the presence of AgPd0.38 on the surface of the catheter helps relieve the host immune responses to bacterial infection and taking into account that free AgPd0.38 had been demonstrated to be able to promote wound disinfection, we in this animal study did not use as many mice for counting CFU per wound as in that examining the performance of free AgPd0.38.
Data exclusions	No data were excluded from the analyses.
Replication	To verify the reproducibility for bacterial and cellular assays, each trial was carried out in triplicate, and the reported results are averages of two independent trials. The observed results show that all the attempts at replication were successful.<br>To verify the reproducibility for animal experiments, the mouse models in each in vivo assay were randomly allocated into different treatment groups with 5 or 6 mice in each group. The observed results show that all the attempts at replication were successful.
Randomization	In each independent assay that involves a bacterial dispersion or a cell culture, the bacterial dispersion or the cell culture was randomly allocated into the control and treatment groups. To ensure randomization, we collected the bacterial or cell culture after it reached appropriate state of cell growth, re-dispersed the as-collected bacteria or cells as planktonic objects into an expected medium (either a buffer or a nutrient medium), and then inoculated an expected and equal amount (usually measured in volume) of the resulting bacterial or cell dispersion into each well of a microplate, and the as-inoculated wells were then allocated to the control and different treatment groups.
Blinding	In laboratory studies, investigators normally are not blinded to group allocation during data collection and/or analysis, despite that, in clinical studies, investigators are required to be blinded to group allocation during data collection and/or analysis.

Reporting for specific materials, systems and methods

We require information from authors about some types of materials, experimental systems and methods used in many studies. Here, indicate whether each material, system or method listed is relevant to your study. If you are not sure if a list item applies to your research, read the appropriate section before selecting a response.

Materials & experimental systems

n/a	Involved in the study
☒	☐ Antibodies
☐	☒ Eukaryotic cell lines
☒	☐ Palaeontology and archaeology
☐	☒ Animals and other organisms
☒	☐ Human research participants
☒	☐ Clinical data
☒	☐ Dual use research of concern

Methods

n/a	Involved in the study
☒	☐ ChIP-seq
☒	☐ Flow cytometry
☒	☐ MRI-based neuroimaging

Eukaryotic cell lines

Policy information about cell lines

Cell line source(s)	The cell lines used in this study are murine macrophage Raw 264.7, murine macrophage Ana-1, murine fibroblast NIH-3T3, and murine breast cancer cell 4T1.<br>All eukaryotic cell lines used in this work were purchased from Cell Bank of the Chinese Academy of Sciences (Shanghai, China).
Authentication	All the bacterial strains and eukaryotic cell lines were used as received without further authentication.
Mycoplasma contamination	The cell lines were not tested for mycoplasma contamination.
Commonly misidentified lines<br>(See ICLAC register)	No commonly misidentified lines were involved in this study.

Animals and other organisms

Policy information about studies involving animals; ARRIVE guidelines recommended for reporting animal research

Laboratory animals	The animals used in this study are ICR mice (female, 6-8-week old). Mice were housed at temperature of 22-25 ºC and 12h/12h dark/light cycle. We don't have information on humidity in mouse housing at hand and did not find the related device that can gauge the humidity in the animal center where our animal studies were carried out .
Wild animals	The study did not involve wild animals.
Field-collected samples	The study did not involve samples collected from the field.
Ethics oversight	The Animal Care and Use Committee at University of Science and Technology of China.

Note that full information on the approval of the study protocol must also be provided in the manuscript.
